# Progress in Surgery

**Published:** 1899-12-23

**Authors:** 


### Progress in Surgery.

### ABDOMINAL SURGERY.

**Gunshot Wounds of the Abdomen.**—These have a special interest at the present time. At the meeting of the British Medical Association at Portsmouth in August last, Colonel Stevenson opened a discussion on the subject. He showed that the improvements in civil practice were scarcely applicable to warfare at present. Bullet wounds of the abdomen in the Crimean and American Civil Wars had a mortality of over 90 per cent. Fæcal extravasation or hæmorrhage is almost certain if a bullet traverses the abdominal cavity, hence operation affords the only rational chance of success. Spontaneous hæmostasis is rare, even when only small vessels are severed; and intestinal wounds are commonly multiple. Hence operation is necessary. The mortality after operation varies from about 55 to 75 per cent., varying with the time which elapses before operation. This indicates that certainly 20 per cent. of cases of abdominal wounds might be saved by adequate and early operation. It must be noted, however, that Senn reported that all the cases died which were operated on in the American army during the war with Spain.[1]

**Ascites Cured by Operation.**—Dr. Drummond and Mr. Rutherford Morrison have introduced a method of providing an anastomotic circulation for the portal blood by producing vascular adhesions between the parietes and the viscera and omentum. Morrison's experience now covers four cases. His first case presented a hob-nailed liver and a much enlarged spleen. The peritoneal cavity was emptied and dried, then the surfaces of the parietal peritoneum, liver, spleen, and exposed intestine were firmly sponged, and the omentum was stitched to the abdominal wall. A glass drainage-tube was inserted into the lower abdomen. The operation was followed by some considerable mental depression. The patient was heard of two years afterwards, and had been provisionally passed as a first-class life for insurance. Another successful case developed a ventral hernia, and was operated on two years afterwards, the result proving fatal. Of two other cases one was successful and another not.[2]

**Surgery of the Liver and Gall-bladder.**—Suture of the liver is now employed both for lacerations and in connection with removal of tumours. Subbotic reports two successful cases of suture for injury. One case was particularly interesting, for a wound of the liver on the right side was complicated by a traumatic diaphragmatic hernia on the left, yet the patient recovered after operation.[3] A similar case of successful hepatorrhaphy for wound is recorded by Pomara.[4]

Seventy-six cases of removal of tumours of the liver have been tabulated by W. W. Keen,[5] including three successful cases of his own. One, more interesting than the rest, involved the removal of the left lobe of the liver, containing a large cancerous mass, by means of the Paquelin cautery. Hæmorrhage was not severe, except from five large veins, which were undermined and ligatured. Finally gauze packing was employed in addition. The weight of the part removed was over a pound and a quarter. Bile was discharged from the wound for 12 days.

The mortality in the 72 cases recorded appears to have been about 15 per cent.

In the same number of the "Annals of Surgery" Dr. Jas. Thompson gives some useful information as to ligature of hepatic substance, and the various measures for mechanical hæmostasis.

Dr. Arthur Bevan has made a number of dissections to determine the best incision for exposing the bile-ducts.[6] He says that the ordinary incisions do not give room enough for exposing the ducts, unless made very long, when there is risk of subsequent hernia. He proposes an incision which answers for exploration and extensive operation, and gives a minimum risk of subsequent hernia. It consists in an *f*-shaped incision through the outer part of the rectus, prolonged laterally as required. The exposure of the parts is ample.

It is not often that a patient twice requires operation for gall-stones, but McBurney records one. At the first operation a stone was removed from the duct by incision of the latter. At the second operation the duodenum was opened and the stone extracted by the duodenal route introduced by that surgeon.[7]

**Surgery of the Stomach.**—Certain cases of simple gastric ulcer should, in the opinion of Tricomi, be treated by gastro-enterostomy for radical cure. Of 50 cases of non-perforating gastric ulcer five died after operation. This shows a mortality of 10 per cent., and it is doubtful whether surgeons will agree with the author as to the advisability of operation.[8]

A very successful case of operation for gunshot wound of the stomach and large intestine was recorded at the New York Medical Society, comprising six perforations in all.[9]

Operations for perforated gastric ulcer are now common, nearly every district having its successful case. It is therefore scarcely necessary to record recent cases. Although plication of the stomach for dilatation is not an established procedure, yet it is gratifying to find such improvement after the operation—performed twice in the same patient—as is recorded by Mr. W. H. Horrocks.[10] The patient was moribund before operation, but left the hospital greatly improved, and with a gain of two stones in weight.

**Pancreatic Cysts.**—Dr. Abbe has shown drawings of three cases illustrating the variable relation of the cyst to the stomach and colon.[11] All three cases were successfully operated upon.

**Intestinal Surgery.**—Ritchie Thomson has described a successful case of volvulus of the sigmoid associated with a hernia into a retrovesical pouch.[12] Ellsworth Eliot records three cases of volvulus, with two recoveries after operations. In one case the patient was operated upon thrice. He recommends that the volvulus be untwisted in all cases, and that the gut be sewn to the parietes to prevent recurrence.[13] Of wounds of the intestine George Woolsey gives a striking instance with 16 perforations; 12 of these were sutured by Lembert's suture, and a piece of gut, including four others, was excised and anastomosed by Murphy's button. This patient recovered, but the bullet was not found. Carl Schlatter has resected nearly two metres of the small intestine, and quotes the opinion that when more than that amount is removed digestion is almost certainly impaired.[14] An ingenious intestinal coupler has been invented by Jacob Frank to replace the Murphy button in anastomosis.[15] It consists of two decalcified bone collars with four needle-hole perforations at the shoulder of each collar, and a piece of rubber tubing to fit inside the collars. The couplers are made in eight sizes. A puckering stitch is passed over the free end of the gut, which is drawn over the collar until the rubber tubing is reached. The union is reinforced by Lambert's sutures. The apparatus has been employed with success, and seeing that the inventor received the congratulations of Dr. J. B. Murphy on the device, it is obvious that commendation from others would be superfluous.

The liability of Meckel's diverticulum to attacks of inflammation resembling appendicitis has been fully established. Magaigne and Blanc report two cases.[16] In one case the diverticulum was found to be inflamed and surrounded by a false membrane, and in the wall was a piece of fish bone.

We may now pass to the surgery of the large intestine. Mr. Makins has described two cases of rupture of the ascending colon, both ending in recovery after operation. One had a complete wound three-quarters of an inch long with hæmorrhages and fæcal extravasation. Makins shows that of 89 cases of abdominal injury at St. Thomas's Hospital one-third were injuries of the intestines. He thinks the determining factor in site of rupture is that the violence should be exerted on the part of the belly cavity, supported by a bony wall posteriorly. Hence intestinal rupture is more common in the lower half of the belly. It is unusual for fæces to be extravasated to any large extent; the mucous membrane of the bowel usually plugs the opening. Severe pain immediately after the injury is not commonly met with, but rigidity and immobility of the abdominal wall are almost constant. The pulse tends to increase in frequency, and diminish in strength. Much effusion of blood should suggest a rent of the mesentery.[17]

Dr. Willy Meyer has brought forward a case of actinomycosis of the cæcum, which recovered after operation and 14 months' treatment with potassium iodide.[18]

Two cases of polyadenoma of the large intestine have been described by Quénn and Landel. They point out the liability of the condition to cancerous transformation.[19]

**Surgery of the Appendix.**—Dr. M. H. Richardson (Boston) gives his experience of over 900 cases. Internal operations were without a death, but the mortality of operation in the acute stage was over 20 per cent. Dr. Richardson took a cautious line of treatment for an American, but Dr. Deaver opposed him, advising early and immediate operation in the majority of cases; but these views were not endorsed by the majority of speakers.[20]

[1] Lancet, Aug. 12, 1899. [2] Lancet, May 27, 1899. [3] Epit. Brit. Med. Jour., Aug. 26, 1899. [4] Ibid., June 24, 1899. [5] Annals of Surgery, Sept., 1899. [6] Ibid., July, 1899. [7] Med. Rec., Sept. 2, 1899. [8] Epit. Brit. Med. Jour., May 13, 1899. [9] Annals of Surgery, Aug., 1899. [10] Ibid., Sept., 1899. [11] Med. Rec., July 22, 1899. [12] Glasgow Méd. Jour., Aug., 1899. [13] Annals of Surgery, July 1899. [14] Epit. Brit. Med. Jour., July 29, 1899. [15] Chicago Med. Rec., July, 1899. [16] Brit. Med. Jour., July 29, 1899. [17] Annals of Surgery, Aug., 1899. [18] Annals of Surgery, July, 1899. [19] Glasgow Med. Jour., Aug. 1, 1899. [20] Med. Rec., Sept. 23, 1899.

